# Microtubular Disruption Alters Intestinal Permeability in Murine Neonatal Anemic Intestine

**DOI:** 10.1155/anem/6607553

**Published:** 2026-05-14

**Authors:** Marie Amalie Balamurugan, Balamurugan Ramatchandirin, Juanitaa George Raj, Arjun Subrramanya, Sayma Azeem, Zainab D. Lawal, Megan M. Ferris, Elizabeth Meister, Krishnan MohanKumar

**Affiliations:** ^1^ Department of Pediatrics, Division of Neonatal-Perinatal Medicine, University of Texas Southwestern Medical Center, Dallas, Texas, 75390, USA, utsouthwestern.edu; ^2^ Department of Biochemistry and Molecular Biology, University of Nebraska Medical Center, Omaha, Nebraska, 68198, USA, unmc.edu; ^3^ Child Health Research Institute, Omaha, Nebraska, USA, chri.org; ^4^ Department of Pediatrics, University of Nebraska Medical Center, Omaha, Nebraska, USA, unmc.edu

**Keywords:** microtubular assembly, neonatal mice, phlebotomy-induced anemia, tubulin

## Abstract

Anemia is a frequent diagnosis in premature and critically ill infants and is caused by phlebotomy, which is essential for medical care. The severity of anemia often correlates with the acuity of illness. We recently showed that severe neonatal anemia causes a “leaky gut” in neonatal mouse pups by altering the structure and function of epithelial adherens junctions through decreased E‐cadherin expression. We hypothesize that microtubules play a major role in maintaining adherens junction integrity. To test this hypothesis, C57BL/6 mice were subjected to timed phlebotomy between postnatal days (P) 2–10 to induce severe anemia (hematocrits 20%–24%). Microtubular assembly was evaluated by electron microscopy, quantitative real‐time polymerase chain reaction (qRT‐PCR), immunohistochemistry, and/or immunoblotting on intestinal tissues, Caco‐2 intestinal epithelial‐like cells, and colonic organoids. The electron micrographs showed loss of microtubules in the anemic intestine and disrupted intestinal epithelial microtubular assembly. Microtubular disruption was associated with decreased expression of the α‐tubulin 4a (tuba4a) subunit. The observed ultrastructural changes that disrupt epithelial barrier functions result from hypoxia‐induced microRNA let‐7e destabilizing tuba4a expression in the anemic intestinal epithelium. In conclusion, phlebotomy‐induced anemia in mouse neonates is associated with a “leaky gut” by the disruption of microtubular assembly.

## 1. Introduction

Anemia is a frequent diagnosis in premature and critically ill infants and is associated with increased morbidity and mortality [1–4]. The severity of anemia correlates with the acuity of an infant’s illness and/or the degree of prematurity, and its etiology may involve multiple factors such as hypoactivity of the bone marrow following postnatal exposure to higher oxygen concentrations, low erythropoietin levels, repeated phlebotomy for laboratory tests, increased red blood cell (RBC) turnover, rapid somatic growth, and nutritional issues such as iron deficiency [5–10].

Severe anemia is clinically important in neonates because its treatment often requires RBC transfusions, which carry their own allergic, hemolytic, and infectious risks. The threshold for RBC transfusions in neonates remains highly variable between centers [11]. This variability is partly due to the fact that most transfusions are prophylactic and administered to maintain hemoglobin levels above predetermined thresholds, rather than to replace actual blood loss [12, 13]. Cognizant of the risks associated with RBC transfusions, many clinicians withhold transfusions in young infants who appear asymptomatic or until the hematocrit drops to levels as low as 20%–22% [14]. However, concerns are also emerging about the possibility of harm from these severe degrees of anemia during critical developmental epochs and whether these cautious transfusion guidelines for young infants, without adequate supporting safety data, may have become far too restrictive [15, 16].

We have recently shown that severe neonatal anemia may increase gut mucosal permeability by altering the structure and function of epithelial adherens junctions through decreased E‐cadherin expression [4]. Considering the important role of microtubules in maintaining adherens junctions [17–19], we designed the present study to investigate whether microtubule loss may also contribute to the loss of adherens junctions in severe anemia. We further investigated whether microRNA let‐7e, which depletes E‐cadherin during severe neonatal anemia, also alters microtubular structure and its functions during severe anemia. To test these hypotheses, we used our neonatal murine model of severe anemia‐related intestinal permeability and examined the microtubules of epithelial cells.

## 2. Methods

### 2.1. Murine Phlebotomy‐Induced Animal Model

Animal studies were approved by the Institutional Animal Care and Use Committee of the University of Nebraska Medical Center and complied with the NIH guide for the care and use of laboratory animals. C57BL/6 mice of both sexes (equal numbers) were studied in control and anemic groups. As described previously [4, 17–20], anemia was induced by facial vein phlebotomy to remove 20 μL of blood per gram body weight on postnatal (P) days of 2, P4, P6, P8, and P10, and an equivalent volume of normal saline was administered subcutaneously. Hematocrits with RBC indices were measured at each phlebotomy; 5 μL blood was diluted 1:20 in Cellpack reagent (Sysmex, Kobe, Japan) and analyzed in the Sysmex XT‐2000iV veterinary hematology analyzer. For tissue studies, animals were typically sacrificed on P11 using CO_2_ inhalation and cervical dislocation, and the proximal and mid‐colon regions were harvested for further studies.

### 2.2. Vibratome Sectioning and Immunostaining

Isolated intestinal tissues were washed with PBS and fixed for 30 min in a 4% formaldehyde solution at room temperature. Fixed intestines were washed in PBS, embedded in 4% UltraPure low‐melting‐point agarose (Invitrogen), and vibratome‐sectioned (Microm HM 650 V; Thermo Fisher Scientific) at 100 μm. The 100‐μm sections were permeabilized for 2 h in PBS containing 1% BSA, 1% DMSO, and 0.2% Triton X‐100 (PBDT) at room temperature and then immunostained for α‐tubulin and tuba4a by overnight incubation at 4°C with either rabbit polyclonal α‐tubulin antibody (cat. no. ab4074, Abcam, Cambridge, MA) or rabbit polyclonal tuba4a antibody (cat. no. PA5‐113679, Thermo Fisher Scientific, Waltham, MA). Secondary staining was performed with Alexa 488‐conjugated antibodies for 30 min (Invitrogen, San Diego, CA). Nuclear staining was obtained with 4′,6‐diamidino‐2‐phenylindole (DAPI) (cat no. D9542; Sigma), and the tissues were then imaged using a Nikon C2 confocal microscope. Images were analyzed using NIH ImageJ.

### 2.3. Immunohistochemistry

Formalin‐fixed paraffin‐embedded tissues were stained as described previously [4, 18, 21]. Briefly, after deparaffinization and antigen retrieval (EZ‐AR Common solution, Biogenex, San Ramon, CA), digestion with Proteinase K (20 μg/mL, 10 min; Promega, Madison, WI), and blocking for 30 min (SuperBlock T20 blocking buffer, Thermo Fisher Scientific), the slides were incubated overnight at 4°C with primary antibodies for α‐tubulin, tubulin α‐chain 4a, occludin, e‐cadherin (cdh1), and phospho‐myosin light chain (MLC) 2 (Ser19) (Cell Signaling, Boston, MA). Secondary staining was performed with Alexa 488 antibodies for 30 min (Invitrogen, San Diego, CA). Coverslips were mounted using a liquid mountant containing DAPI (ProLong Gold Antifade Mountant, Thermo Fisher Scientific), and the tissues were imaged using a Nikon C2 confocal microscope.

### 2.4. RT‐Quantitative PCR

As previously described [4, 18], we used a standard SYBR green method to measure mRNA expression. Primers were designed using the Beacon Designer software (Bio‐Rad, Hercules, CA).

### 2.5. Western Blots

Immunoblots were performed as previously described [22] to measure tubulin α‐chain 4a, MLC‐2, phospho‐MLC2 (Ser19), and β‐actin in intestinal tissue or Caco‐2 cells.

### 2.6. Transmission Electron Microscopy (TEM)

Tissue samples from the proximal colon were fixed in 2% glutaraldehyde and then in 1% osmium tetroxide and processed for TEM (JEOL 1400 digital transmission electron microscope) as previously described [4]. For microtubular imaging, thin sections were photographed to analyze 8 epithelial cells in tissue samples from each mouse.

### 2.7. Caco‐2 Cells and Colonic Organoids

Caco‐2 cells (cat. no. HTB‐37, American Type Culture Collection) were cultured in DMEM (cat. no. 10566016, Thermo Fisher Scientific) with 10% fetal calf serum (cat. no. 16000044, Thermo Fisher Scientific) in 6‐well plates. In some experiments, we transfected Caco‐2 cells with let‐7e‐5p inhibitor (cat. no. 4464084, Thermo Fisher Scientific) and cultured them under hypoxic conditions (1% oxygen). Mouse colonic crypts were cultured using established methods as previously described [4].

### 2.8. MicroRNA and Let‐7e Inhibitor Assay

MicroRNAs were measured by using the standard method described previously [4]. In vitro cultured Caco2 cells and colonic organoids were transfected with anti‐tuba4a siRNA (Thermo Fisher Scientific) or the let‐7e‐5p mimic, using the Lipofectamine RNAiMax reagent (Thermo Fisher Scientific), and microRNA binding was measured as previously described [4].

### 2.9. Let‐7e Inhibitor In Vivo Study

Mouse pups were treated intraperitoneally at each phlebotomy from P4–P10 with scrambled miR‐inhibitor or with specific miR‐let‐7e‐5p inhibitor (25 mg/kg body wt; miRCURY LNA let‐7e‐5p inhibitor, cat. no. 339203, Qiagen). Plasma endotoxin levels were measured according to a previously described method [4] in the following groups: (1) control mice treated with scrambled miR inhibitor, (2) control mice treated with let‐7e‐5p inhibitor, (3) anemic mice treated with scrambled miR inhibitor, and (4) anemic mice treated with let‐7e‐5p inhibitor. Tuba4a expression was measured by RT‐qPCR in the intestines from each group on P11.

### 2.10. Statistical Analysis

Statistical analysis was performed using GraphPad Prism software, Version 9 (GraphPad software, La Jolla, CA). Differences were considered significant at *p* < 0.05.

## 3. Results

### 3.1. Severe Anemia Associated With Loss of Microtubules in the Intestine

**FIGURE 1:**
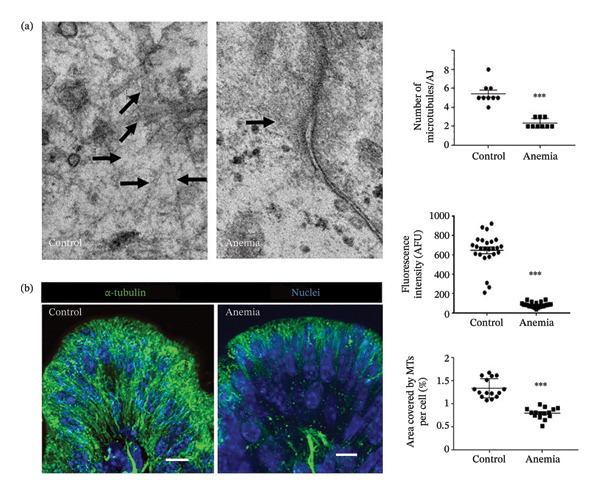
Severe anemia associated with loss of microtubules in the intestine. (a) Representative transmission electron micrographs (85,000x) of colonic epithelium from control and anemic pups. Unlike control, epithelial cells in anemic colon showed few microtubules associated with the adherens junctions. Scatter dot plot (means ± SE) on right summarizes the number of microtubules per adherens junction. (b) Fluorescence photomicrographs (360x) of control and anemic neonatal colon showing immunoreactivity for α‐tubulin in epithelial cells. The anemic colon showed microtubular disassembly, particularly near the plus (+) ends of microtubules at the basolateral membrane. Scale bar = 10 μm. Scatter plots (means ± SE) on the right summarize the fluorescence intensity (top) and area covered by microtubules in cells (bottom), respectively. Data represent 5 mice/group.

We have recently described a preclinical murine model of phlebotomy‐induced anemia and RBC transfusion‐related NEC, in which severe anemia increases intestinal permeability by disrupting epithelial adherens junctions’ protein E‐cadherin. In this study, we reexamined our tissue specimens using transmission EM at higher magnification to further define the ultrastructural abnormalities in the adherens junctions. The anemic intestine showed a marked reduction in the number of microtubules associated with the epithelial adherens junctions (Figure 1(a)). These findings were confirmed by immunohistochemistry staining for α‐tubulin (Figure 1(b)). The originating or minus (−) ends of the microtubules near the apical membrane seemed relatively preserved, but the plus (+) ends toward the basolateral membrane were severely depleted and replaced by punctate staining for monomeric tubulin. These findings were suggestive of microtubular disassembly.

### 3.2. Decreased Expression of Tuba4a Can Explain the Loss of Microtubules

To investigate the mechanism(s) underlying the loss of microtubules in the anemic intestine, we used RT‐qPCR to measure mRNA expression of genes involved in microtubule anchoring to adherens junctions [23] (Figure 2(a)), regulation of microtubule assembly (including the plus‐end binding proteins) [24] (Figure 2(b)), and structural components of microtubules (Figure 2(c)). We found a significant reduction in the expression of a single gene, tubulin α‐chain 4a (Tuba4a). We proceeded to confirm these findings at the protein level. The anemic intestine showed decreased tuba4a immunoreactivity in epithelial cells (Figure 2(d)). Western blots on exfoliated epithelial cell lysates confirmed the loss of tuba4a at the protein level (Figure 2(e)).

FIGURE 2Decreased expression of Tuba4a can explain the loss of microtubules: (a) bar diagrams (means ± SE) show fold change in mRNA expression of genes involved in anchoring of microtubules to adherens junction, (b) gene regulators of microtubular assembly, and (c) genes that play a structural role in microtubule formation. Anemia was associated with decreased tubulin‐α, chain 4a (Tuba4a). *N* = 8 mice per group. Mann–Whitney *U* test, ^∗∗^ *p* < 0.01. Plekha7 = pleckstrin homology domain‐containing A7, Camsap3 = calmodulin‐regulated spectrin‐associated protein‐3, Kifc3 = kinesin family member C3; Map = microtubule‐associated protein, Mapt = Map‐tau, Mapre = microtubule‐associated protein, RP/EB family, Dctn = dynactin, clip = cytoskeleton‐associated protein glycine‐rich domain‐containing linker protein, Clasp = cytoplasmic linker‐associated protein, Tuba = tubulin‐α, Tubal = tubulin‐α‐like, Tubb = tubulin β, Tubd = tubulin‐δ, tube = tubulin‐ε, Tubg = tubulin‐γ, Atat = alpha‐tubulin N‐acetyltransferase, Tbc = tubulin folding cofactor, and Tbcel = tubulin folding cofactor E like. (d) Fluorescence photomicrographs (360x) of the control and anemic neonatal colon show decreased Tuba4a immunoreactivity in epithelial cells in anemic colon. Scale bar = 10 μm. (e) Western blots show Tuba4a expression in exfoliated epithelial cells isolated from control and anemic colon. The bar diagram above (means ± SE) shows densitometric ratios to β‐actin. *N* = 6 (3 mice/group; experiment run twice).

**Figure:**
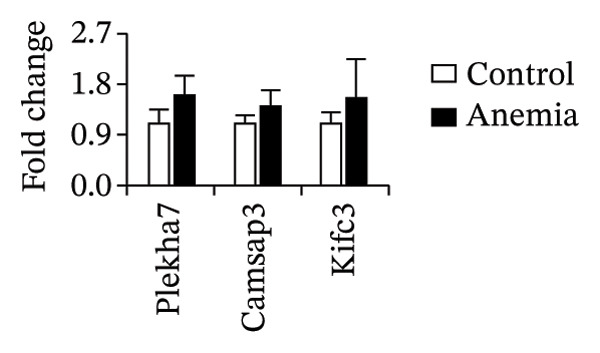
(a)

**Figure:**
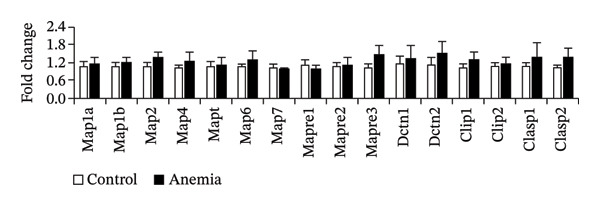
(b)

**Figure:**
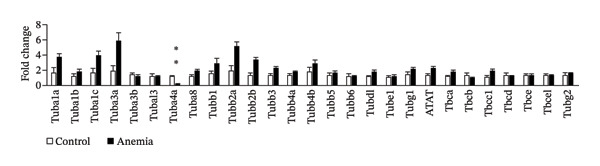
(c)

**Figure:**
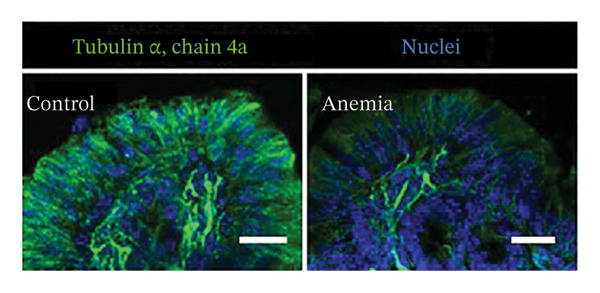
(d)

**Figure:**
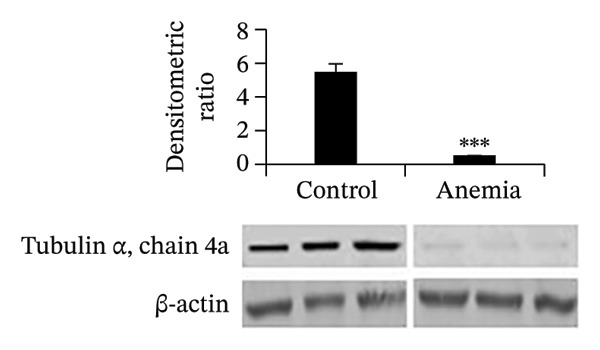
(e)

### 3.3. Loss of Microtubules Alters Adherens Junctions in the Anemic Intestine

To confirm whether tuba4a is required for microtubule assembly in gut epithelial cells, we used RNA interference to knock down tuba4a first in Caco‐2 cells and then in colonic organoids derived from mouse crypts [25]. Tuba4a knockdown reduced the microtubule abundance, confirming that tuba4a is an essential component of microtubules in these cells (Figure 3(a)). Tuba4a knockdown reduced transepithelial electrical resistance (TEER; Figure 3(b)) but did not change E‐cadherin expression at the protein level (Figure 3(c)). Immunostaining indicated some redistribution of E‐cadherin (and occludin) away from the cellular contacts (Figure 3(d)).

FIGURE 3Loss of microtubules alters adherens junctions in the anemic intestine. (a) Fluorescence photomicrographs (1200x) showing immunoreactivity for α‐tubulin in Caco‐2 cells (left) and cultured colonic organoids (right) transfected with scrambled (control) vs. specific anti‐Tuba4a siRNA. (b) Tuba4a knockdown in Caco‐2 cells interrupted the normal development of transepithelial electrical resistance (TEER). Line diagrams summarize TEER across Caco‐2 monolayers transfected with scrambled (control) vs. specific anti‐Tuba4a siRNA. Data represent 6 replicates/group. (c) Western blots show that Tuba4a knockdown did not alter E‐cadherin expression. Bar diagram above (means ± SE) shows densitometric values normalized to β‐actin. *N* = 6 (3 mice/group; experiment run twice). (d) Fluorescence photomicrographs of Caco‐2 cells treated with anti‐Tuba4a or scrambled (control) siRNA, immunostained for occludin (top) and E‐cadherin (bottom). Tuba4a knockdown led to redistribution of occludin and E‐cadherin away from cell–cell contacts. Intensity profiles on the right trace the fluorescence intensity along the transverse white line drawn across the photomicrographs. The concentrations of occludin and E‐cadherin near cellular contacts, seen as repeated spikes in the intensity profiles, were lost following Tuba4a knockdown. Magnification 250x. Data represent 6 replicates per group. (e) Fluorescence photomicrographs (800x) of control and anemic neonatal colon showing increased immunoreactivity for phosphorylated myosin light chain (Ser19) in epithelial cells in anemia. Scale bar = 10 μm. (f) Western blots performed on exfoliated epithelial cells from control and anemic intestines show phosphorylated (Ser19) and total regulatory myosin light chain (MLC) and β‐actin. Bar diagrams above (means ± SE) summarize the densitometric ratios, normalized to β‐actin. Data represent *n* = 6 replicates (3 mice/group, 2 independent runs).

**Figure:**
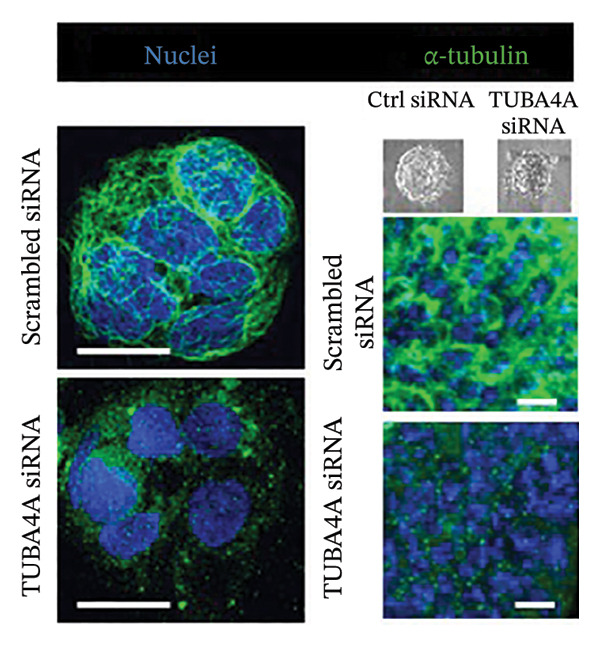
(a)

**Figure:**
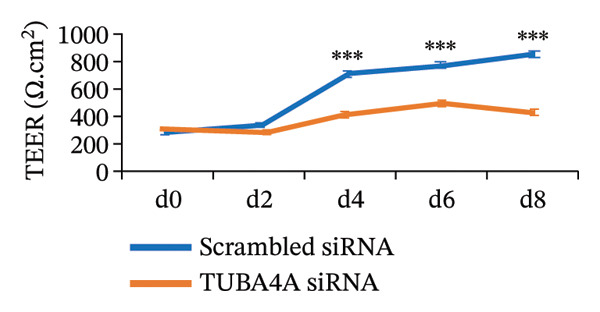
(b)

**Figure:**
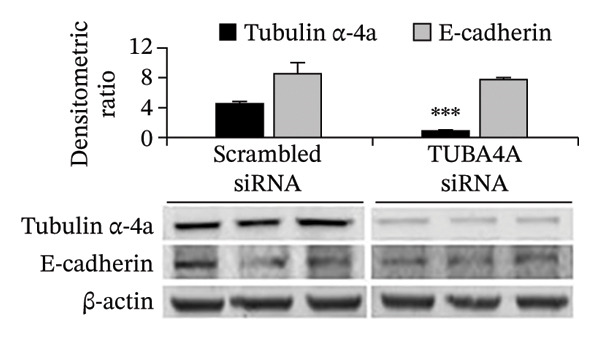
(c)

**Figure:**
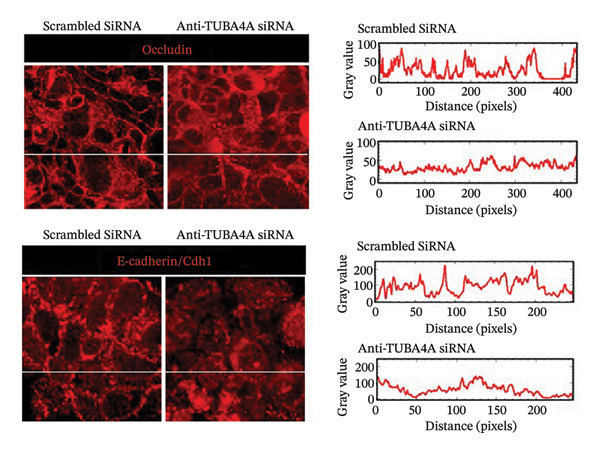
(d)

**Figure:**
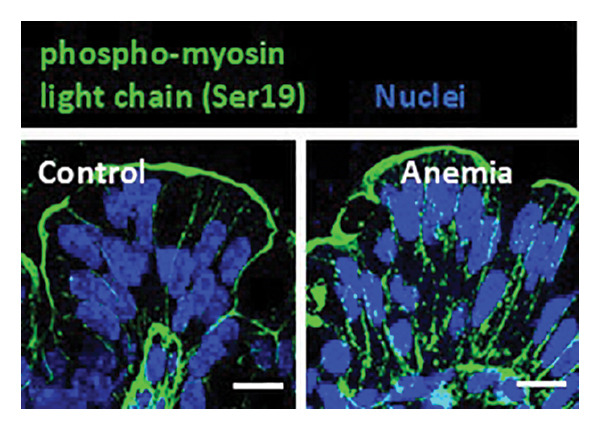
(e)

**Figure:**
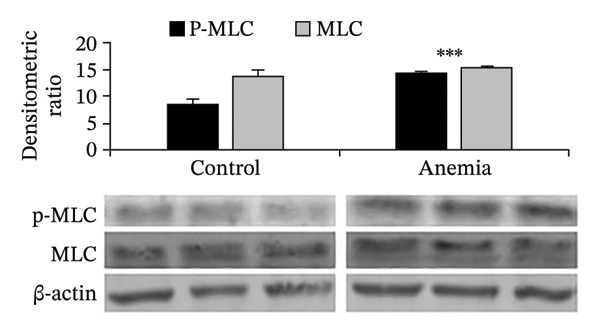
(f)

These findings were explained by cytoskeletal changes; existing information indicates that microtubule disruption can promote MLC phosphorylation in nonmyocyte cell lineages, and the resulting cytoskeletal contraction can augment paracellular permeability [26]. The anemic intestine showed increased phospho‐MLC (Ser19) in epithelial cells (Figure 3(e)), as confirmed by western blots of exfoliated epithelial cells (Figure 3(f)).

### 3.4. MicroRNA Let‐7e Binds and Destabilizes Tuba4a mRNA in the Anemic Intestine

Previously, we had identified disparate mechanisms to explain increased intestinal permeability in anemia—abnormalities in the adherens junctions due to loss of E‐cadherin by miR let‐7e‐5p [4]. We first analyzed the 3′‐untranslated region (3′UTR) sequences of tuba4a in silico to predict microRNA‐binding sites using the online tools STarMir and miRWalk2.0 (from http://sfold.wadsworth.org/cgi-bin/starmirtest2.pl). Predicted binding configurations of let‐7e‐5p with the 3′UTRs of tuba4a are depicted in Figure 4(a). Tuba4a mRNA has several alternatively spliced isoforms; transcripts *a* and *b* encode full‐length proteins, whereas variants *c*, *d*, *e*, *g*, and *h* produce truncated proteins. Variant *f* is an unspliced transcript of uncertain significance (Figure 4(b)). Direct measurement of these mRNA variants by RT‐qPCR did not show differential regulation of full‐length *vs*. truncated Tuba4a transcripts in anemia and therefore did not support alternative splicing as a likely mechanism of decreased tuba4a expression (Figure 4(d)). The two full‐length transcripts *a* and *b* did show greater reduction in anemia than transcript variants *c and dg-h*. Variants *a* and *b* both display substantial 3′‐UTR. Therefore, we next asked whether these UTRs could be a target for a hypoxia‐responsive microRNA. In support of these findings, we also observed increased levels of miR‐99b‐5p and miR‐125a‐5p in anemia (Figure 4(e)), two other microRNAs that share a promoter with let‐7e and are located in the same cistronic cluster [27].

FIGURE 4MicroRNA let‐7e binds and destabilizes Tuba4a mRNA in the anemic colon. (a) Let‐7e binding sites on the 3′‐UTRs of Tuba4a mRNA are conserved across species (from https://www.targetscan.org/vert_80//). (b) Schematic shows the alternatively spliced mRNA variants of murine Tuba4a (from https://www.ncbi.nlm.nih.gov/ieb/research/acembly/av.cgi?db=mouse%26term=tuba4a%26submit=Go). The amplicons’ sites in RT‐qPCR are indicated by red double‐headed arrows. Variants *a* and *b* encode full‐length proteins, whereas *c, d, e* (not depicted due to incomplete sequence), *g*, and *h* represent truncated proteins. Variant *f* is an unspliced transcript of uncertain significance. (c) Schematics show the predicted binding structures of murine and human let‐7e‐5p to murine and human tuba4a sequences, respectively (from http://sfold.wadsworth.org/cgi-bin/starmirtest2.pl). (d) Severe neonatal anemia was associated with decreased expression of both full‐length and truncated mRNA splice variants of tuba4a. Bar diagram (means ± SE) shows fold change in full‐length (variants *a, b*) and truncated (*c,* *g* *, h*) mRNA splice variants of tuba4a in control and anemic neonatal colon. Splice variants *d* and *e* were not expressed in the neonatal colon. *N* = 8 mice/group. (e) Bar diagrams (means ± SE) show the fold change in the expression of miR‐99b and miR‐125a in the anemic intestine (vs. control), after normalization to sno55. These two microRNAs are located in the same cistronic cluster as let‐7e. *N* = 6 mice per group. Mann–Whitney *U* test, ^∗∗^ *p* < 0.01. (f) Hypoxia‐mediated suppression of tuba4a mRNA in Caco‐2 cells was reversed by a let‐7e inhibitor. Bar diagram (means ± SE) shows the expression of tuba4a in Caco‐2 cells in normoxic versus hypoxic conditions, in cells transfected with a let‐7e miRNA inhibitor versus miRNA control. Let‐7e inhibition reversed the effects of hypoxia on tuba4a expression (*p* < 0.001). (g) Let‐7e binds the 3′‐UTR of Tuba4a mRNA. Caco‐2 cells were transfected with a luciferase reporter containing the 3′‐UTR sequences of Tuba4a mRNA, along with let‐7e‐5p. Bar diagram shows decreased luciferase activity 24 h after transfection; data summarize 6 replicates. (h) Let‐7e reduced mRNA stability of Tuba4a mRNA. Line diagrams show means ± SE fold change in the Tuba4a mRNA, measured by RT‐qPCR. ^∗^ *p* < 0.05; ^∗∗^ *p* < 0.01; data summarize 6 replicates. Mann–Whitney *U* test. (i) Let‐7e overexpression suppresses Tuba4a expression in Caco‐2 cells. Western blots show bands that are immunoreactive for Tuba4a in Caco‐2 cells overexpressing either the control miRNA (miR‐1) or let‐7e‐5p. Bar diagrams above (means ± SE) depict densitometric ratios to β‐actin. *N* = 6 (3 replicates/group in 2 runs). (j) Let‐7e‐5p overexpression in Caco‐2 cells disrupts the development of transepithelial electrical resistance (TEER). Line diagrams show serial TEER measurements in Caco‐2 monolayers transfected with miR‐1 vs. let‐7e‐5p. *N* = 6 replicates/group. (k) Fluorescence photomicrographs (1000x) show immunoreactivity for tuba4a in Caco‐2 cells (left) and colonic organoids (right) overexpressing miR‐1 vs. let‐7e‐5p. Scale bar = 15 μm. *N* = 6 per group. (l) Bar diagrams (mean ± SE) show mRNA expression of tuba4a expression, hematocrits (%), hemoglobin (g/L), mean corpuscular volume (MCV; fL), mean corpuscular hemoglobin (MCH; pg), mean corpuscular hemoglobin concentration (MCHC; g/L), and red cell distribution width (RDW; fL) in (1) P10 control mice treated with scrambled miR inhibitor (*n = *6), (2) control mice treated with miR let‐7e‐5p inhibitor (*n = *6), (3) anemic mice treated with scrambled miR inhibitor (*n = *6), and (4) anemic mice treated with miR let‐7e‐5p inhibitor (*n = *6). Tuba4a expression was restored in anemic mice treated with miR let‐7e‐5p inhibitor. Mann–Whitney *U* test, ^∗∗∗^ *p* < 0.001.

**Figure:**
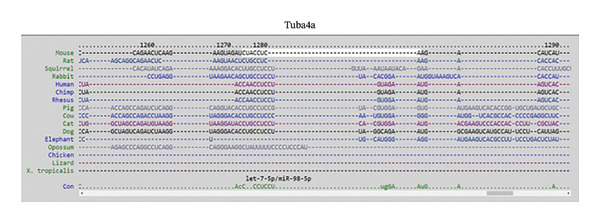
(a)

**Figure:**
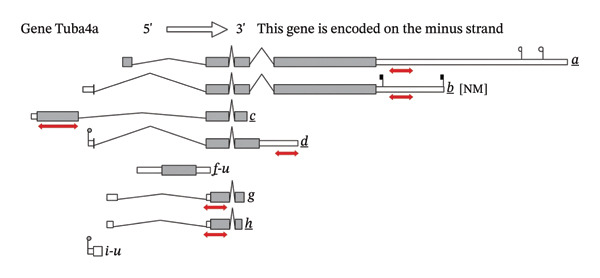
(b)

**Figure:**
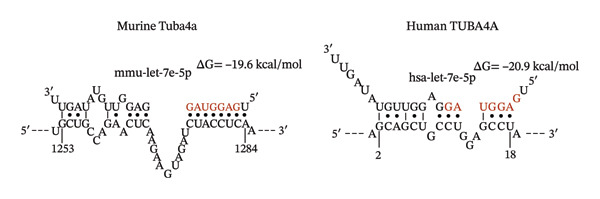
(c)

**Figure:**
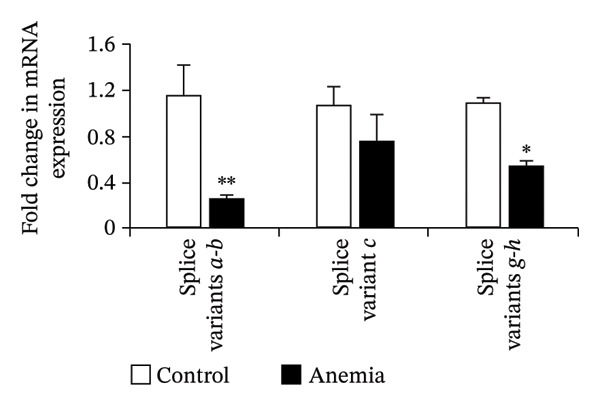
(d)

**Figure:**
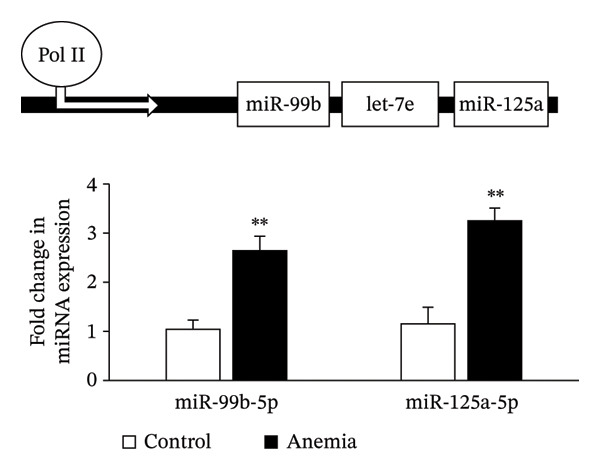
(e)

**Figure:**
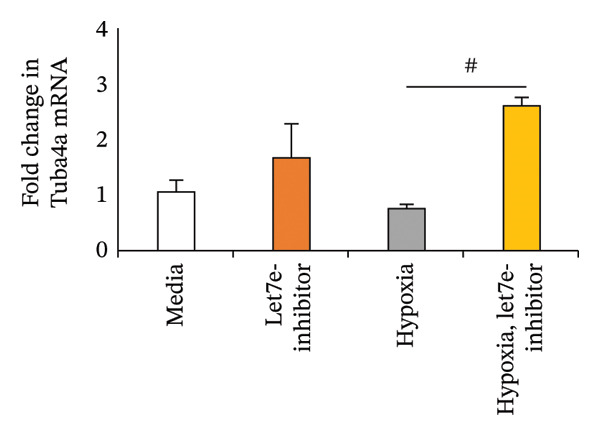
(f)

**Figure:**
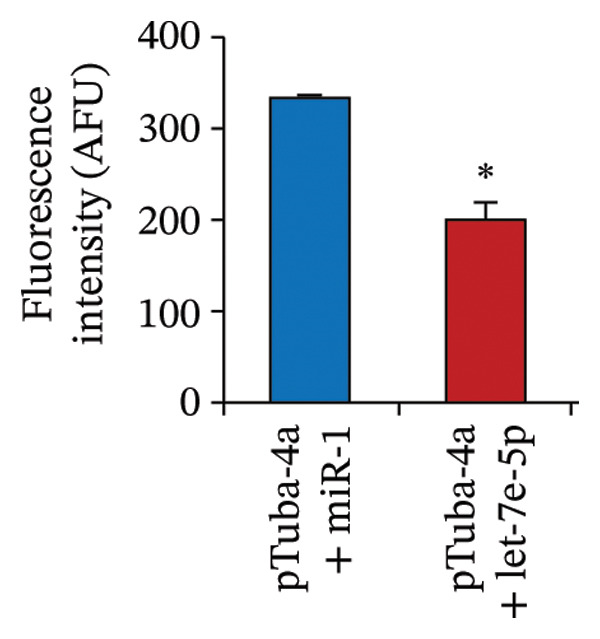
(g)

**Figure:**
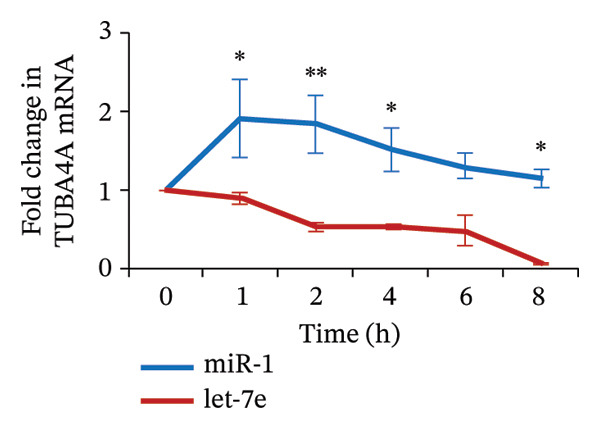
(h)

**Figure:**
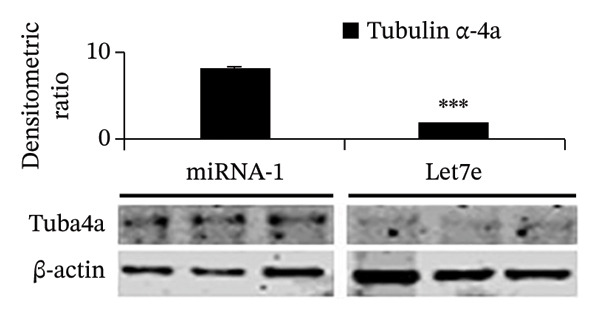
(i)

**Figure:**
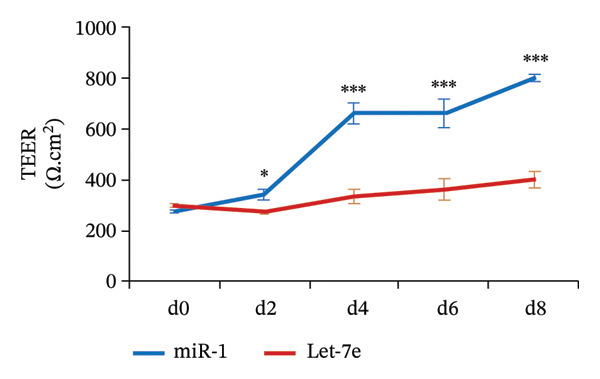
(j)

**Figure:**
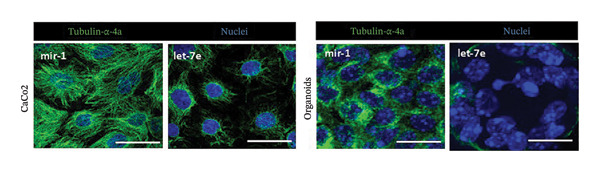
(k)

**Figure:**
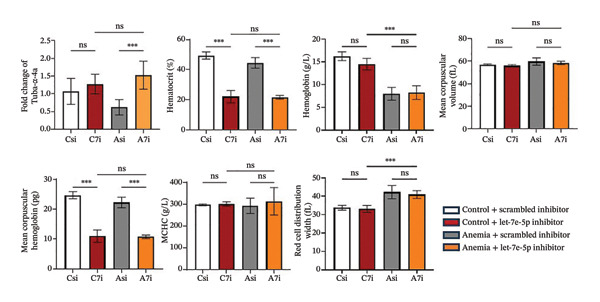
(l)

### 3.5. MicroRNA Let‐7e Degrades Tuba‐4a mRNA in the Anemic Intestine

To determine the role of increased let‐7e expression on suppressed tuba‐4a expression during hypoxic conditions, we grew Caco‐2 cells under normal and hypoxic conditions (17% or 1% oxygen, respectively). Under both conditions, cells in some plates were transfected with a let‐7e inhibitor. Hypoxia increased let‐7e‐5p expression and suppressed in Caco‐2 cells, effects reversed by the let‐7e inhibitor (Figure 4(f)). To confirm let‐7e‐5p binding to the 3′‐UTR of Tuba4a mRNA, we transfected Caco‐2 cells with luciferase reporters containing these 3′‐UTR sequences, along with let‐7e‐5p (or miR‐1, scrambled control). Co‐transfection with let‐7e‐5p reduced luciferase activity in pTuba‐4a constructs, indicating direct binding to and destabilization of the UTR sequences (Figure 4(g)). Consistently, Caco‐2 cells transfected with miR‐let‐7e (or miR‐1), and with actinomycin D added to block ongoing transcription, showed increased turnover of these transcripts at serial time points, by RT‐qPCR (Figure 4(h)).

### 3.6. Let‐7e‐5p Suppresses Tuba4a and Increases Intercellular Permeability and Microtubule Disassembly in Epithelial Cells

Western blots of Caco‐2 cells transfected with let‐7e confirmed decreased Tuba4a at the protein level (Figure 4(i)). Let‐7e overexpression in Caco‐2 monolayers also reduced TEER (Figure 4(j)), indicating interrupted development of epithelial junctions. Finally, let‐7e overexpression in Caco‐2 cells (Figure 4(k)—left) and in colonic organoids derived from neonatal mouse crypts (Figure 4(k)—right) suppressed Tuba4a expression and disrupted cellular junctions and microtubules. Tuba‐4a mRNA expression in anemic pups treated with the miR let‐7e‐5p inhibitor was restored to levels similar to those in control pups (Figure 4(l)) with no significant changes in hematocrit, mean corpuscular volume (MCV), mean corpuscular hemoglobin (MCH), and mean corpuscular hemoglobin concentration (MCHC), but hemoglobin and RDW levels were increased in anemic mice treated with miR let‐7e‐5p inhibitor.

## 4. Discussion

We observed that severe neonatal anemia leads to significant disruption in intestinal microtubular assembly, which is critical for maintaining epithelial barrier function. Specifically, the loss of tubulin α‐chain‐4a (Tuba4a) in the intestinal epithelium led to microtubule disassembly, thereby impairing the structural integrity of adherens junctions. Microtubules, which are essential for the stability and organization of epithelial cells, were notably depleted at their growing plus (+) ends, typically located near the basolateral membrane. This depletion weakens the intestinal barrier, increasing permeability to luminal antigens and potentially harmful bacterial translocation. Interestingly, although microtubules in polarized epithelial cells are typically anchored to adherens junctions [17], we found that disassembly was most pronounced near the plus ends rather than at the junctions themselves, with no notable changes in the genes responsible for anchoring microtubules to adherens junctions. The role of tuba4a in microtubular integrity in neonatal anemia is novel, marking the first report of this gene’s involvement in the gastrointestinal tract. Given that tuba4a is one of nine paralogous α‐tubulins in mammals [31], its distinct lack of a C‐terminal tyrosine residue may affect the tyrosination–detyrosination cycle and, consequently, microtubular stability [28]. The specific function of this isotype within the broader framework of microtubule assembly and whether different tubulin isotypes serve distinct roles remain areas of ongoing research.

Additionally, we identified that the microRNA let‐7e plays a pivotal role in destabilizing Tuba4a expression during anemia. Our analysis indicated that let‐7e, which was upregulated in response to hypoxia in anemic neonates, directly binds to the 3′‐UTR of Tuba4a mRNA, leading to its degradation. We previously described that microRNA let‐7e‐5p was constitutively expressed at higher levels in the mouse pups than in adults and was further upregulated in severe anemia. Members of the let‐7 microRNA family exhibit contrasting response patterns during hypoxia. Thus, *let-7g*, *let-7e*, and *let-7i* were identified as hypoxia‐inducible, whereas *let-7a, c, d, e, f*, and *g* levels decreased during hypoxia exposure. The caveat that the findings were reported in different cell types and by different groups may explain why let‐7e has been reported as both hypoxia‐inducible and decreased during hypoxia exposure [29]. Let‐7e is known to play important roles in gut epithelial differentiation, and its high constitutive expression in neonates was expected, given its developmental role [30]. Hypoxia is a known inducer of let‐7e in both endothelial cells and cancer, where these microRNAs derepress vascular endothelial growth factor expression and promote angiogenesis [31]. By demonstrating the interaction between let‐7e and Tuba4a, we were able to establish a mechanistic link between hypoxia and microtubule disassembly in the neonatal intestine. Further work is needed to elucidate the specific mechanisms driving let‐7e induction in severe neonatal anemia.

To further explore the therapeutic implications, we employed a let‐7e inhibitor in anemic neonatal mice, which successfully restored Tuba4a expression and microtubular stability, but showed no changes in most RBC indices. This intervention resulted in improved epithelial barrier function, as evidenced by reduced permeability [4] and enhanced junction integrity. These findings suggest that targeting let‐7e may represent a viable therapeutic strategy to mitigate the adverse effects of anemia on intestinal permeability. Additionally, our in vitro models using Caco‐2 cells and colonic organoids confirmed that Tuba4a depletion directly contributes to microtubule instability and barrier dysfunction. These results reinforce the importance of microtubules in maintaining epithelial integrity and underscore the therapeutic potential of microRNA inhibition in this context.

## 5. Conclusions

We have demonstrated a novel mechanism of microtubular disruption that adds to the already known mechanisms whereby disruption of epithelial adherens junctions in the intestine causes “leaky gut” during severe neonatal anemia. Our study suggests that targeting pathways such as microtubule dynamics could offer novel therapeutic opportunities and that transfusion thresholds for anemic neonates may need to be revisited in light of these insights.

## Author Contributions

K.M. designed the study, and K.M. and M.A.B. wrote the manuscript; M.A.B., B.R., J.G.R., A.S., S.A., Z.D.L., M.M.F., E.M., and K.M. performed key experiments and data analysis. All the authors contributed to the manuscript.

## Funding

This study was funded by the National Institutes of Health (NIH) with awards HL163043, HD105880, and HD116950 (to K.M.).

## Disclosure

All authors approved the manuscript.

## Ethics Statement

The authors have nothing to report.

## Conflicts of Interest

The authors declare no conflicts of interest.

## Data Availability

The data that support the findings of this study are available from the corresponding author upon reasonable request.
